# Dataset of experimental measurements for the Orion beam structure^☆^

**DOI:** 10.1016/j.dib.2021.107627

**Published:** 2021-11-23

**Authors:** Rafael de Oliveira Teloli, Pauline Butaud, Gaël Chevallier, Samuel da Silva

**Affiliations:** a UNESP - Universidade Estadual Paulista, Faculdade de Engenharia de Ilha Solteira, Departamento de Engenharia Mecânica, Av. Brasil, 56, Ilha Solteira, SP 15385-000, Brazil; b Univ. Bourgogne Franche-Comté, FEMTO-ST Institute, CNRS/UFC/ENSMM/UTBM, Department of Applied Mechanics, 24 chemin de l’Epitaphe, Besancon 25000, France

**Keywords:** Orion beam, Frictional damping, Experimental practices, Contact mechanics, Bolted joint

## Abstract

This data article comprises experimental data to investigate the nonlinear dynamic behavior of the Orion beam structure, which consists of two duraluminum beams assembled by bolted joints. To retain contact on a small area between both beams, this new lap-joint configuration proposes contact patches at each bolt connection. The Orion beam suggests an assembly configuration that associates bolts dedicated to ‘static’ functions and to those to perform ‘damping’ functions. This ensures a significant increase in the structural damping without degrading the structural stiffness. Experiments have been performed on the laboratory ple Vibration and Acoustic, located at FEMTO-ST Institute, CNRS/UFC/ENSMM/UTBM, Department of Applied Mechanics, 24 chemin de lEpitaphe, 25000 Besanon, France.

This data aim to provide the geometrical step and all the experimental measurements performed on our lap-joint for several excitation amplitudes and tightening torques, as far as possible and with the degree of uncertainties of the measurements. By doing so, we intend to provide experimental data, as precise and reliable as possible, which are required to progress on the numerical modelling of the dry friction damping in assembly structures.

This Data in Brief article is an additional research item alongside the following paper published in the Mechanical Systems and Signal Processing journal: R. O. Teloli, P. Butaud, G. Chevallier and S. da Silva, Good practices for designing and experimental testing of dynamically excited jointed structures: the Orion beam.

**Specifications Table**

**Table:** 

Subject	Mechanical Engineering
Specific subject area	Nonlinear vibration, dry friction, nonlinear damping
Type of data	*.mat files and ASCII files
How data were acquired	There are two datasets (namely dataset #1 and dataset #2). Regarding dataset #1, the excitation of the structure was conducted by an eletromagnetic Modal Shop Shaker (Model K2004E01). The excitation force was measured with a load cell. Regarding dataset #2, the structure was subjected to base excitation driven by a permanent magnetic shaker TIRA (TV Model 51120), whereas the acceleration at the base was monitored by a triaxial accelerometer PCB (Model 356A4). In both datasets, a Polytec vibrometer PSV−500 with a 3D scanning laser is used to measure the velocity of the Orion beam. The setup also includes a National Instruments acquisition system composed of a CompactDAQ Chassis (NI cDAQ - 9134), C-Series Sound, and Vibration Input Module (NI-9263), and C-Series Voltage Output Module (NI-9234). Each bolt’s torque value was checked by a Lindstorm MA500-1 torque wrench.
Data format	Raw
Parameters for data collection	In both datasets, the tightening torque was monitored for different values, such as 80, 60, 30, 20 and 10 cNm. For dataset #1, excitation values were controlled using the force value measured by the load cell, while for dataset #2, acceleration at the base was controlled for different amplitude levels.
Description of data collection	Dataset #1 contains multiple measure points that span the whole structure for white noise type input with low level of excitation amplitude to obtain the linear response of the structure. To highlight nonlinearities present in the system due to dissipative effects, a single-point measurement was considered for step-sine input. The 3rd and 6th bending modes were investigated under this condition. Dataset #2 considers a white noise input and the response data were acquired through a single measurement point. Several levels of base acceleration were tested and the response of the first six bending modes were acquired under these conditions.
Data source location	Institution: Data obtained from the laboratory *pôle Vibration and Acoustic*, located at FEMTO-ST Institute, CNRS/UFC/ENSMM/UTBM, Departament of Applied Mechanics. City/Town/Region: 24 chemin de l’Epitaphe, 25000 Besançon. Country: France
Data accessibility	Repository name: Both datasets are available on Mendeley’s Repository Data identification number: 10.17632/p4fg6snh3r.1 Direct URL to data: https://data.mendeley.com/datasets/p4fg6snh3r/1
Related research article	R. O. Teloli, P. Butaud, G. Chevallier, S. da Silva, Good practices for designing and experimental testing of dynamically excited jointed structures: The Orion beam, Mechanical Systems and Signal Processing, 163(2022) 108172. https://doi.org/10.1016/j.ymssp.2021.108172

## Value of the Data

• This article provides the geometrical step, the numerical mesh, and the experimental measurements performed on the Orion beam for several levels of excitation amplitude and tightening torques. Thus, the structure design and experimental tools addressed in this work could be useful to characterize the frictional dissipation induced by the micro-slip that occurs at the joint interfaces.
• The datasets aim to benefit the scientific community that investigates the dynamics of jointed structures, as well as readers interested in experimental practices applied to nonlinear systems, modelling and designing of damping links. Based on the experimental measurements performed on the Orion beam, one can have the possibility to compare numerical models with experimental responses to evaluate the predictive capability of nonlinear dynamic models, apply identification methodologies, or even propose condition monitoring algorithms to monitor variations in the tightening torque.
• From dataset #1, it is possible to have an estimate of the frequency response curves and, from these, the backbone curves that can give an insight into the nonlinear behavior of the vibrating mode that stresses the region around the lap-joint in a more pronounced way. We suggest using the dataset #2 to develop structural health monitoring techniques for detecting bolt tightening loss, since the tests include white-noise excitation considering different levels of tightening torque and excitation amplitudes. Finally, both datasets can be used for further insights on new control techniques in jointed structures for vibration reduction by combining vibration amplitude attenuation and bolt preload.

## 1 Data Description

### 1.1 Dataset #1

The dataset aims to supply the readers with all the raw data needed to obtain the same results used in the article [1]. This dataset is provided as supplementary data in a Matlab format and ASCII format. From these data, one can obtain Figs. 9(a), 10, 12, 13 and 16 of the article [1], which correspond, respectively, to the complete scan of the Orion beam in the glued condition, the frequency response curves referring to the third bending vibration for different excitation levels, repeatability of the Orion beam after complete disassembly for seven experimental runs, repeatability of the Orion beam without disassembly, and frequency response curves for the sixth vibration mode considering several levels of tightening torque and force amplitudes. A description on each dataset is given as follows:

• Complete 3D scan:

The file provides access to the complete 3D scan of the Orion beam considering the contact patches perfectly glued. With this data, the reader is able to construct the experimental mesh of the beam from 195 sensing points, which span the structure’s surface, as well as the frequency response curve estimated after applying a low-amplitude white noise excitation (63.45 mN RMS) band-limited between 10 Hz and 2000 Hz.

• Frequency response curves corresponding to the 3rd bending mode:

One can select among the following conditions: 10, 50, 100, 150, 200 mN as excitation force, 20 and 80 cNm as tightening torque. The data correspond to step-sine tests starting at 280 Hz up to 300 Hz with a frequency increment of 0.3901 Hz.

• Measurement repeatability considering complete disassembly of the Orion beam:

One can select among the following conditions: 10 and 250 mN as excitation force, 20 and 80 cNm as tightening torque and seven different repetitions (1,2,3,4,5,6 and 7). The repetition vector corresponds to the measurements for seven experimental runs in frequency response curves. These measurements are related to the 6th bending mode.

• Measurement repeatability without the complete disassembly of the Orion beam:

One can select among the following conditions: 10 and 250 mN as excitation force, 20 and 80 cNm as tightening torque and four different repetitions (1,2,3 and 4). The realization vector corresponds to the measurements for four experimental runs in frequency response curves. These measurements are related to the 6th Bending Mode.

• Frequency response curves from the 6th bending mode:

One can select among the following conditions: 10, 50, 100, 150, 200 and 250 mN as excitation force, 10, 20, 30 and 80 cNm as tightening torque. The data correspond to step-sine tests starting at 1660 Hz up to 1780 Hz with a frequency increment of 1 Hz. Further details related to the acquisition process are present in [1] and a Matlab file is available in [2] to aid in post processing the data.

Regarding the frequency response curves, Table 1 and Table 2 show a general description about the variables present in each data file:

**Table 1:** List of data and the corresponding description.

Name	Description
ChannelAcc	Channel 1 on the acquisition board
ChannelForce	Channel 2 on the acquisition board
ChannelVoltage	Channel 3 on the acquisition board
ChannelVel	Channel 4 on the acquisition board
Torque	Tightening torque condition
Control	Channel selected for amplitude control
Fe	Frequency vector
Fe_n	Structure format contaning complex amplitudes of acceleration [m/s${}^{2}$], force measured with the load cell [N], voltage supplied on the shaker amplifier [V] and velocity [m/s] for each frequency increment

**Table 2:** List of data related to *Dataset* #2 and the corresponding description.

Name	Description
time	Time vector
N	Number of samples
T	Test duration
data	Base acceleration in X, Y and Z directions and velocity measured at the free-end of the beam.

Considering more specifically the equipment used to acquire vibration measurements, one has the following list of devices:

• To measure the velocity of response of the structure, a Polytec vibrometer PSV-500 with 3D scanning laser was used;
• The excitation force applied to the structure was driven by an eletromagnetic Modal Shop Shaker (Model K2004E01), which was connected to the Orion beam by a threaded nylon rod stinger;
• The force transmitted by the shaker was monitored by a load cell PCB 288D01;
• The tightening torque at the central and external bolts (for reference, please see [1]) was checked using a Lindstorm MA500-1 torque wrench;
• The resulting force from the tightening torque applied to the bolts was measured by a Futek LTH300 donut load cell;
• The setup also includes a National Instruments acquisition system composed of a C-Series Sound and Vibration Input Mode (NI-9234) and a CompactDAQ Chassis (NI cDAQ - 9134).

### 1.2 Dataset #2

The dataset is intended to supply readers with raw data related to white noise type excitation tests performed on the Orion beam. This dataset is provided as supplementary data in a Matlab format. Three identical Orion beam specimens were tested. For each specimen, the white noise excitation was band-limited between 10 Hz and 2000 Hz. Regarding this dataset, the structure was subjected to base excitation such that the base acceleration level was controlled to different RMS values (1 m/s${}^{2}$, 4m/s${}^{2}$, 8m/s${}^{2}$ and 12 m/s${}^{2}$). Tests were performed assuming a sampling frequency of 25.6 kHz and 262,144 samples (time duration 10.24 s).

To evaluate the influence of the tightening torque on the response of the structure, the central bolt is maintained fully tightened with a torque of 80 cNm at all experimental measurements. On the other hand, five different levels of torque are applied to the external bolts: 80 cNm, 60 cNm, 30 cNm, 20 cNm, and 10 cNm.

**Fig. 1:**
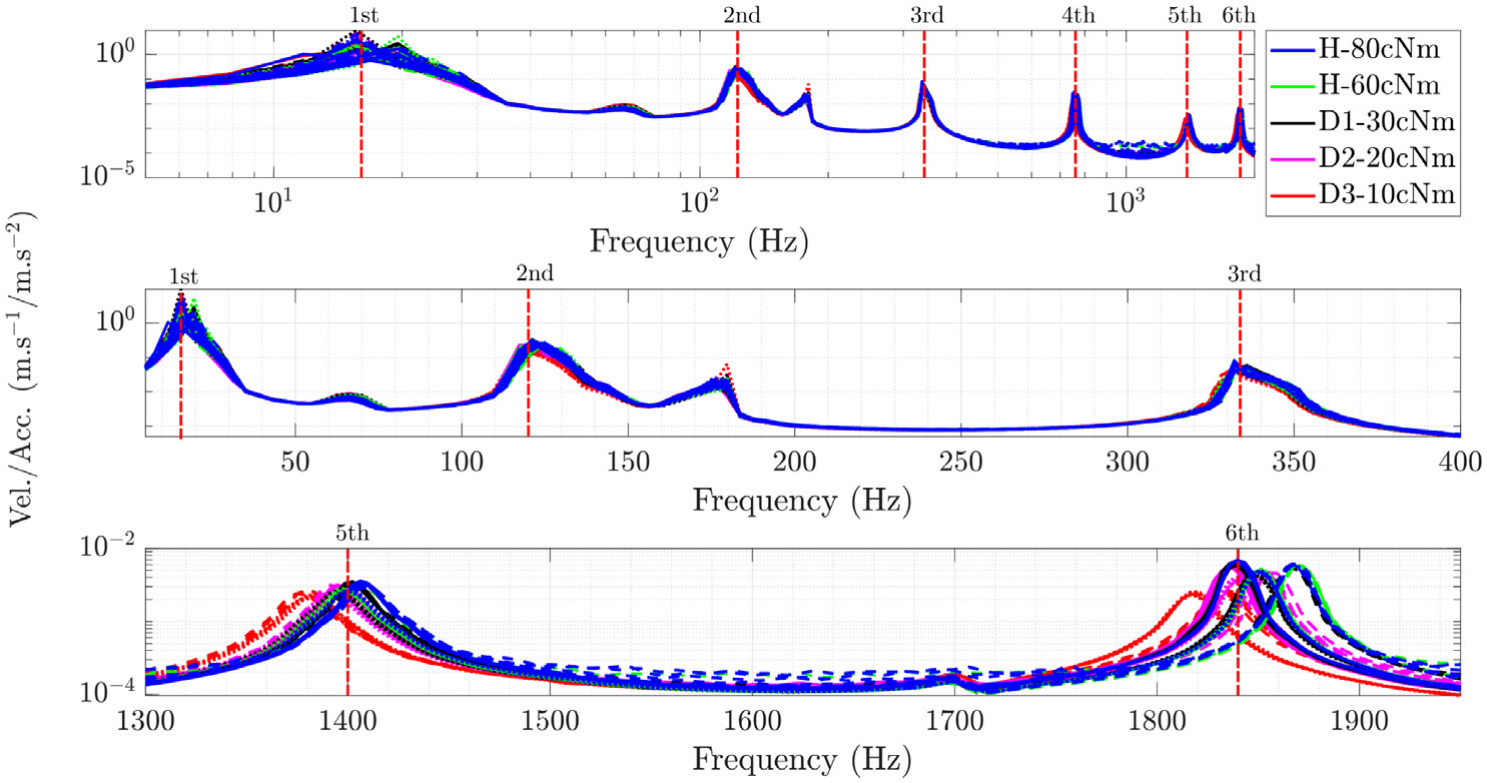
Transmissibility plot with emphasis on the structural conditions. Dashed lines −− indicate the frequency ranges in which the bending modes are. Different lines of the same color indicates different assemblies; **H** indicates healthy state and **D** is damaged state.

In each specimen, the experimental campaign are performed on three sets of measurements after complete assembly and disassembly of the Orion beam, repeated on different days to obtain a total of 12 realizations for each torque level. The use of this data is suggested for proposing/testing techniques for detecting torque loss. *A priori*, torque conditions at 80 cNm and 60 cNm can be assumed to be healthy conditions; torque values at 30 cNm, 20 cNm and 10 cNm can be assumed to be damage conditions due to the loss of connecting properties. Fig. 1 presents the transmissibility spectrum between the velocity measured at the free end of the beam (output) and the base acceleration (input) for several tightening torque and considering a base excitation with amplitude of 4 m/s${}^{2}$. The curves presented in Fig. 1 were obtained considering the $H_{1}$ estimator, hanning window and 32 averages. A Matlab file is available in [2] to aid in post processing the data.

**Fig. 2:**
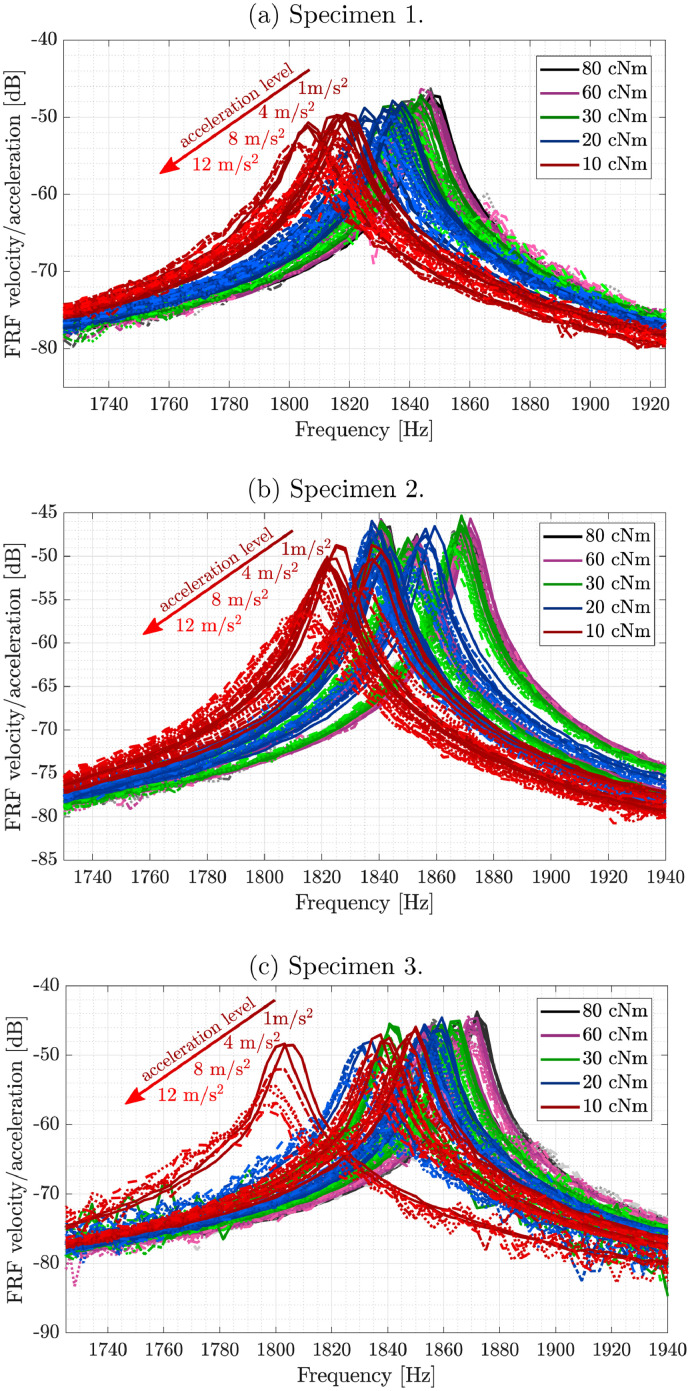
Transmissibility plot with emphasis on different levels of base acceleration around the 6th bending mode (1730 Hz and 1940 Hz). Torques are distinguished by different colors, whereas multiple excitation levels are distinguished by different line types and gradient color.

Fig. 2 depicts the frequency response curves around the 6th bending mode for several base acceleration levels (1 m/s${}^{2}$, 4 m/s${}^{2}$, 8 m/s${}^{2}$ and 12 m/s${}^{2}$) and considering three different specimens of the same beam design. The curves were also obtained by using the $H_{1}$ estimator, hanning window and 32 averages. Table 3 shows a general description about the variables present in each data file.

## 2 Experimental Design, Materials and Methods

The Orion beam consists of two assembly duraluminum beams (2790 kg/m^3^ and 73 GPa of density and Young’s modulus, respectively) with dimensions of 200 × 30 × 2 [mm] and connected by three M4 bolts spaced along a length of 30 mm. A washer is placed under each bolt and nut. On one of the beams, there are contact patches at each bolt connection, consisting of a square of 12 x 12 mm with an extra thickness of 1 mm. The full description of the benchmark along with 3D drawings of the structure are discussed in Teloli et al. [1]. In both datasets, the same protocol for assembling the structure is followed:

• To guarantee the alignment between both beams, two-axis are inserted in the external patches;
• The central bolt is fully tightened;
• Both axes are removed and the external bolts are thus tightened.

Each bolt’s torque value was checked by a Lindstorm MA500-1 torque wrench. The central bolt is always tightened with a preload of 80 cNm, whereas different torque levels were applied to the external bolts. The selection criteria for these torque values are discussed in [1].

For all measurements, to ensure as close to a clamped boundary condition as possible, the excitation tests were conducted with a length of the beam with the contact patches completely fixed and screwed into a massive block. The details presented up to this point are common to both datasets; the specific details of each experimental design are presented below.

### 2.1 Dataset #1

To characterize how the tightening of the external bolts influences the dry friction effects on the system’s response, step-sine tests around resonant frequencies to isolate nonlinear modes were conducted. To minimize the force drop-off phenomenon of the excitation force in the vicinity of the resonances during step-sine tests, a real-time feedback controller was used to control the root mean square (RMS) values of the applied force. The controlled RMS force values were 10, 50, 100, 150, 200 and 250 mN.

An eletromagnetic Modal Shop Shaker (Model K2004E01) is attached 30 mm from the clamped end of the beam by a threaded nylon rod stinger. The applied force was measured by a load cell (Model PCB 288D1). To measure the response data of the Orion beam when subject to dynamic testing, a contact-less Polytec 3D scanning laser PSV-500 was used. The frequency response curves of the data reported above were acquired with a single-point measurement located at 221.5 mm from the clamped end on the beam’s centerline.

The experimental protocol to obtain the frequency response curves is briefly described as follows: firstly, acquisition parameters and excitation settings related to increment and frequency range, desired excitation amplitude, initial supply voltage of the shaker, time duration of the test and sampling frequency were defined. Then, a script algorithm of the feedback controller is executed until the convergence. The full description on how to perform the experimental measurements of the data presented hereby, the analysis and processing of the acquired signals, parameters used during experimental tests and the algorithm executed by the feedback controller are extensively covered in [1].

### 2.2 Dataset #2

**Fig. 3:**
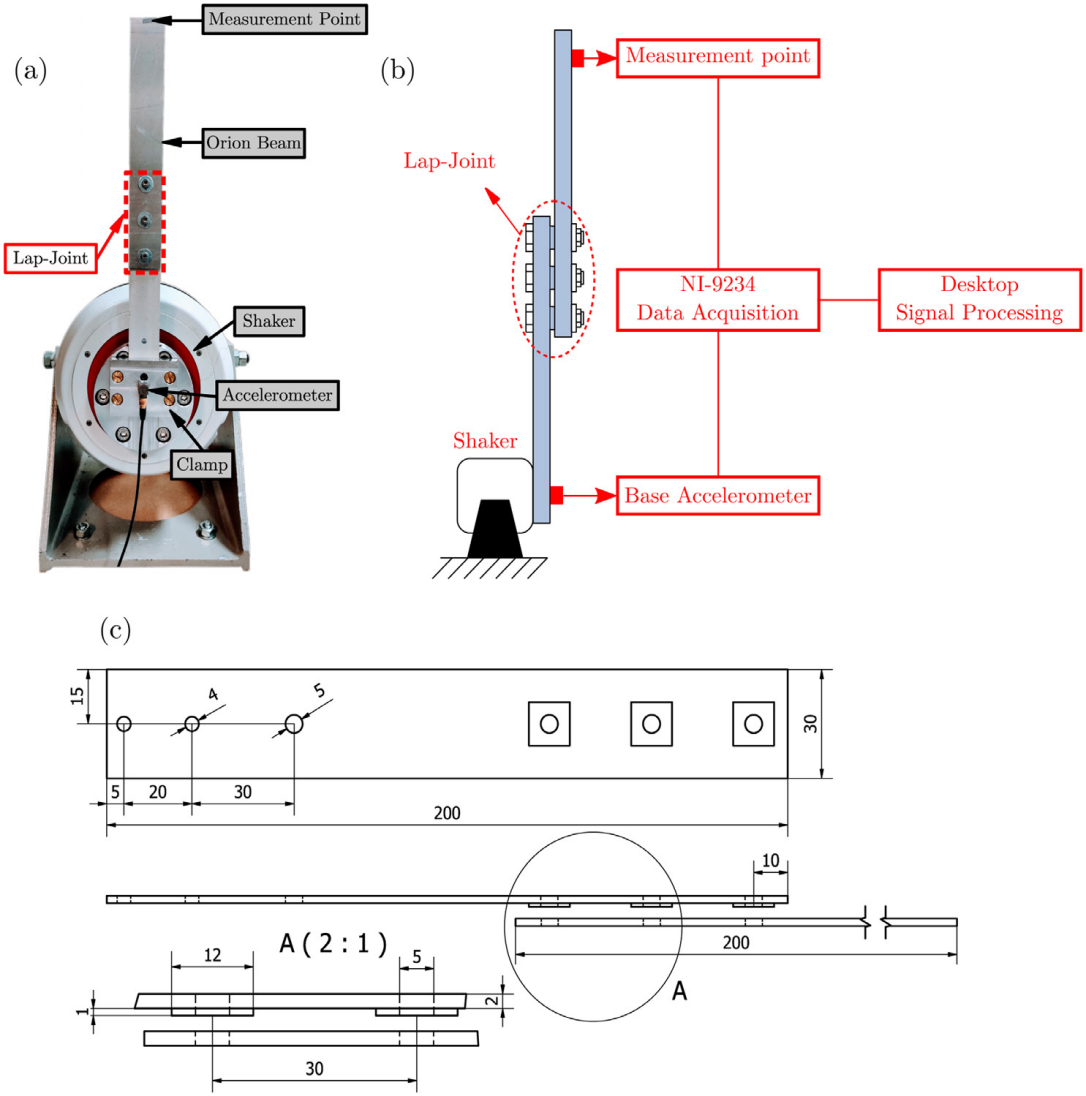
Experimental setup - The Orion beam. (a) Experimental configuration; (b) Schematic representation of the structure with experimental apparatus; (c) Lap-joint drawings CAD (lengths are in mm) - 2 beams, two clamp holes Ø4 mm, one excitation hole Ø5 mm, three contact patches.

The experimental setup is shown in Fig. 3, and it consists of two assembly duraluminum beams in a cantilever configurations under base excitation. A 40 mm length of the beam with the contact patches is screwed to a solid aluminum block, which is the base of the structure. This block is directly connected to the shaker. To avoid effect of gravity as well as the presence of torsional modes on the vibrational behavior of the beam, the structure is positioned vertically with its neutral line perpendicular to the ground. Fig. 3(b) depicts a schematic representation of the experimental setup, whereas Fig. 3(b) represents the CAD model of the Orion beam.

The base motion is driven by a permanent magnetic shaker TIRA (TV Model 51120), which excites the structure considering a white-noise Gaussian input. The control of the RMS level of the base acceleration was done using the same real-time feedback controller proposed in Teloli et al. [1]. A Polytec vibrometer PSV-500 with a 3D scanning laser was used to measure the velocity at the Orion beam’s free end. In contrast, the acceleration at the base is monitored by a triaxial accelerometer PCB (Model 356A4). Base acceleration and velocity measurements are used to estimate the transmissibility spectrum. The setup also includes a National Instruments acquisition system composed of a CompactDAQ Chassis (NI cDAQ - 9134), C-Series Sound, and Vibration Input Module (NI-9263), and C-Series Voltage Output Module (NI-9234).

## CRediT authorship contribution statement

**Rafael de Oliveira Teloli:** Writing – original draft, Visualization, Investigation, Validation, Software. **Pauline Butaud:** Formal analysis, Data curation, Writing – review & editing, Investigation, Software. **Gaël Chevallier:** Project administration, Supervision, Investigation, Writing – review & editing, Conceptualization. **Samuel da Silva:** Supervision, Writing – review & editing.

## Declaration of Competing Interest

The authors declare that they have no known competing financial interests or personal relationships which have, or could be perceived to have, influenced the work reported in this article.

## References

[1] Teloli R.O., Butaud P., Chevallier G., Silva S.d. (2022). Good practices for designing and experimental testing of dynamically excited jointed structures: The orion beam. Mech. Syst. Signal Process..

[2] R. O. Teloli, P. Butaud, G. Chevallier, S. d. Silva, The orion beam dataset, mendeley data, v1, 2021, 10.17632/p4fg6snh3r.1. PMC8633856 34877390

